# Nutrition knowledge and bone health: development and validation of the ‘NutriBone’ questionnaire for Italian adult women

**DOI:** 10.1017/S1368980026102444

**Published:** 2026-04-10

**Authors:** Silvia Callegaro, Kyriaki Apergi, Francesca Scazzina, Daniela Martini, Donato Angelino, Alice Bongrani, Alice Rosi, Giovanni Passeri

**Affiliations:** 1 Department of Food and Drug, University of Parma, Italy; 2 Department of Food, Environmental and Nutritional Sciences (DeFENS), Università degli Studi di Milano, Italy; 3 Department of Bioscience and Technology for Food, Agriculture and Environment, University of Teramo, Italy; 4 Department of Medicine and Surgery, University of Parma, Italy

**Keywords:** Osteoporosis, Woman, Nutrition knowledge, Questionnaire, Bone health, Health literacy

## Abstract

**Objective:**

This study aimed to develop and validate a questionnaire assessing the nutrition knowledge (NK) of Italian adult women regarding the relationship between diet, lifestyle and bone health.

**Design:**

A thirty-item questionnaire in Italian was developed by experts based on a literature review. Participants completed the questionnaire twice, with a 2–4 week gap between the two administrations. During the initial administration, weight and height were recorded using a mechanical scale and a stadiometer, while bone mineral density (BMD) of the lumbar spine (L1-L4), femoral neck and total femur were assessed via dual-energy X-ray absorptiometry (DXA).

**Setting:**

Centre for Metabolic Bone Diseases at the Parma University Hospital, from January 2022 to June 2024.

**Participants:**

Women aged 45–75 years old, native Italian speakers, undergoing DXA at the Centre participated.

**Results:**

The sample included 295 women with a median age of 63 years (interquartile range 11·5). The questionnaire demonstrated good internal consistency (Cronbach’s alpha = 0·698) and high temporal stability (R = 0·810, *P* = 0·002), effectively differentiating between individuals with and without a nutritional background. Regression analysis indicated negative associations between NK score and age (*β*1 = –0·130, *P* < 0·001) and BMI (*β*1 = –0·193, *P* < 0·001).

**Conclusions:**

The NutriBone questionnaire is a valid and reliable tool for evaluating NK related to bone health in Italian adult women undergoing DXA, with potential for future research applications.

Osteoporosis is a major non-communicable disease nowadays, and its prevalence is expected to increase markedly in the future, partly due to the increased life expectancy in both developed and developing countries^(1)^. The disease is characterised by low bone mass and microarchitectural deterioration of bone tissue, resulting in greater bone fragility and a consequent increased fracture risk^(2)^. This condition is present in older subjects, both males and females, particularly in postmenopausal women, as the rapid decline in oestrogen levels that occurs during menopause accelerates bone loss^(3)^.

In Italy, fragility fractures are a major public health concern, affecting approximately 1·5–2 % of adults over the age of fifty on annual basis^(4)^. The associated costs amount to around 9·45 billion euros annually, representing approximately 6 % of the national healthcare budget^(5)^. In this context, primary prevention strategies focusing on modifiable risk factors, such as lifestyle, diet and physical activity, are essential for maintaining health, enhancing quality of life and reducing economic burdens, among postmenopausal women and older subjects.

Dietary behaviours, including food choices, are influenced by numerous environmental and individual factors, including socioeconomic and psychosocial factors such as knowledge, beliefs and perceptions about nutrition and health^(6)^. Food and nutrition knowledge (NK) have been shown to impact diet quality^(6)^ and serve as a linking factor between socioeconomic status and dietary habits^(7)^. NK refers to an understanding of concepts related to nutrition and health, including the association between diet and health and diet and disease, the nutritional content of foods, and the current dietary guidelines and recommendations^(8)^.

Despite the recognised importance of NK, studies specifically focusing on its relationship with bone health are limited. One study in postmenopausal women showed that educational interventions to enhance NK led to improved eating habits and reduced spinal bone loss compared to a control group^(9)^. Another study found that participation in osteoporosis education programmes resulted in a higher level of knowledge regarding the disease and increased dietary calcium intake after three months in adults over 50 years of age with a history of fractures^(10)^.

Specific questionnaires, appropriately designed and tailored to collect information on food and NK in the target population, are commonly used to assess NK. In Italy, for example, a general NK questionnaire has been validated in an adult population over 35 years of age^(11)^. Other NK questionnaires have been validated for other target populations such as Italian university students^(12)^ or for specific aspects such as sports NK in Italian early adolescents^(13)^. To the best of our knowledge, no NK questionnaire focusing on the relationship between nutrition and bone health has been validated in the Italian population. Therefore, the aim of this study was to develop and validate a questionnaire designed to assess the knowledge regarding the relationship between diet, foods, lifestyle habits and bone health in a cohort of Italian adult women.

## Materials and methods

### Participants

All patients admitted for dual-energy X-ray absorptiometry (DXA) at the Centre for Metabolic Bone Diseases (Osteoporosis Center) at the Parma University Hospital from January 2022 to June 2024 were invited to participate in the study. Inclusion criteria were being female, aged between 45 and 75 years, and native Italian speaker.

### Study design

All participants received a detailed explanation of the study protocol from a trained investigator and provided a signed informed consent. To ensure confidentiality, participants were identified by alphanumeric codes. On the day of the DXA scan, participants were asked to provide sociodemographic and educational information, including age, menopausal status, use of vitamin D or calcium supplements, use of osteoporosis medication, level of education and whether they had a background in nutrition or health sciences (i.e. had completed vocational secondary school, college or university that include specific courses in nutrition, food science or health education subjects or had worked in these fields). A telephone number was also collected to facilitate the second administration of the NutriBone questionnaire.

The NutriBone questionnaire was administered to each participant under standardised conditions on two separate occasions. The first administration was done orally, by a trained investigator, on the day of DXA scan, while the second administration was carried out via telephone 2–4 weeks later, a time frame considered sufficient to avoid recall bias while short enough to minimise changes in the attributes under assessment^(14)^.

After the initial administration of the questionnaire, participants underwent a DXA scan to collect bone health parameters. Concurrently, weight and height were measured using a mechanical scale (SECA, 761, Germany) and a stadiometer (Holtain Ltd, Crymych, Dyfed). BMI was then calculated as weight (in kg) divided by the square of height (in metres).

### Development and validation of NutriBone nutrition knowledge questionnaire

The selection of items for the questionnaire was based on the existing literature on NK and food literacy. The authors, all experts in nutrition and bone health, selected questions from an existing questionnaire concerning osteoporosis preventive behaviours^(15)^ and from other pertinent scientific sources to cover all relevant topics^(16–18)^. This process resulted in a pool of eighty-four items. Each author independently evaluated all items and indicated whether each was considered ‘essential’ or ‘not essential’ for inclusion. The content validity ratio was then calculated for each item, which reduced the number of final items to thirty. For each question, there were three possible answers plus an additional ‘*I don’t know*’ option, to avoid missing data or random responses. Correct responses were awarded +1 point, while incorrect or ‘*I don’t know*’ answers received 0 points.

Internal consistency and test–retest reliability were assessed to validate the questionnaire. In addition, the answers from the first administration were used to perform item analysis (item difficulty and item discrimination index) and to explore the construct validity of the questionnaire by comparing participants with and without a nutritional/health background.

### Dual-energy X-ray absorptiometry

To compare the nutritional knowledge with bone mineral density (BMD) and assess the potential diagnosis of densitometric osteoporosis, DXA scans were performed at the lumbar spine and proximal femur (total femur and femoral neck). BMD was measured by DXA technique using the HOLOGIC Discovery A system, following standard protocols. Lumbar spine (L1–L4), total femur and femoral neck BMD were expressed as T-scores^(19,20)^. DXA scans were performed by a certified radiology technician and then analysed by a dedicated physician. According to the WHO criteria (WHO, 1994), participants were classified as normal if spine and/or femoral BMD was above −1 sd, osteopenic if BMD was between −1 and −2·5 sd, and osteoporotic if BMD was below −2·5 sd ^(21)^.

### Sample size and statistical analysis

Based on existing literature, a sample size of 200 respondents is fair and 300 is good^(22)^. Other authors suggested that the required sample size for questionnaire validation should be six to ten times the number of questions^(23)^. As the NK questionnaire contained thirty items, the total number of participants required for the validation study was conservatively estimated to be between 180 and 300.

The normality of the distribution of variables was assessed using the Kolmogorov–Smirnov test and histograms. Continuous variables were presented as mean and sd for normally distributed data or as the median and interquartile range (Q1–Q3) for non-normally distributed data. Categorical variables were reported as proportions (%) compared to the total. Demographic differences between participants with and without a nutritional background were analysed using the χ^2^ test for categorical variables and the Welch *t* test for independent samples or Wilcoxon rank sum test (with continuity correction) for continuous variables depending on their distribution. No imputation for missing values was performed.

Item-level analysis identified questionnaire items that positively or negatively affected reliability when removed. The item difficulty index was calculated based on the frequency of correct answers for each question, with cut-offs between 0·1 and 0·9, indicating items that were not useful if less than 10 % or more than 90 % of participants answered correctly^(14,24)^. Item discrimination was evaluated using a point-biserial correlation between the score for each question and the total score, with values > 0·2 to indicate an acceptable correlation^(14,25)^.

The internal consistency of the NK score was evaluated using Cronbach’s alpha for Likert scale items measuring attitudes and the Kuder–Richardson 20 for binary scale items assessing knowledge and practice dimensions^(14)^. Cronbach’s alpha values greater than 0·6 were considered acceptable^(14,26)^.

Construct validity was examined using the Welch *t* test for independent samples to compare NK scores between participants with and without a nutritional background at the first administration (t1).

An ANCOVA was conducted to compare the total NK scores between participants with and without a nutritional/health background, while controlling for the effect of educational level as a covariate.

Reliability (test–retest time stability) was assessed using Pearsons’s correlation and the intraclass correlation coefficient (2·1) to compare NK scores between the initial (t1) and follow-up (t2) administrations. Higher intraclass correlation coefficient values indicated greater reliability.

Univariable regression analysis was conducted to investigate potential associations between NK scores and the variables examined controlled for age. To examine whether adding a quadratic age term improved model fit, we compared a quadratic model (age + age²) with a linear model using an ANOVA. Polynomial models were fitted to account for non-linearity, with model selection based on the Akaike Information Criterion and likelihood-ratio test of nested models. Model assumptions, including the examination of outliers and their influence, were assessed using plots of standardised residuals and Cook’s D statistics.

All statistical analyses were performed using R version 4.1.2 (R Core Team, 2021), with RStudio version 1.4.1717 (RStudio Team, 2020). Both software packages are available from the R Foundation for Statistical Computing and RStudio, PBC, respectively.

## Results

### Characteristics of participants

Participants’ baseline characteristics are presented in Table 1 for the total sample and by background group. A total of 404 individuals were invited to complete the questionnaire, of whom 295 were eligible as they met the study’s inclusion criteria. Most of the excluded individuals were older than 75 years. Among the eligible participants, 15 % had a nutritional/health background and 85 % had no nutritional/health background. The median age was 63 years (IQR 11·5) overall, with no significant difference between the nutritional/health background and no nutritional/health background groups (*P* = 0·412). There were no significant differences between groups for weight, height and BMI, the latter being within normal ranges. Educational level differed significantly between groups (*P* < 0·001), with more participants in the nutritional/health background group having completed a degree or postgraduate studies. Conversely, menopausal status, use of nutritional supplements and use of osteoporosis medication did not differ significantly between groups (*P* > 0·05). Bone density results for the spine, femoral neck and total femoral regions showed no significant differences between groups (*P* > 0·05).

**Table 1.** Sample characteristics (*n* 295)

	Total participants (*n* 295)		Participants with nutritional/health background (*n* 43)		Participants without nutritional/health background (*n* 252)		*P*-value
	Mean or Median	sd or IQR	Mean or Median	sd or IQR	Mean or Median	sd or IQR	
Age (years)	63	57–68·5	63	55–67	62·5	57–69	0·412
Body weight (kg)	60·0	53·0–67·0	62·0	52·0–66·0	60·0	53·0–67·0	0·534
Height (cm)	160·0	7·0	161	7·0	160·0	7·0	0·148
BMI (kg/m^2^)	23·3	20·9–26·6	23·5	20·9–25·6	23·2	20·9–26·8	0·957
	*n*	%	*n*	%	*n*	%	
Educational level							< 0·001
Middle school or below	88	29·8	6	14·0	82	32·5	
High school diploma	133	45·1	17	39·5	116	46·0	
Graduated/Post-graduated	74	25·1	20	46·5	54	21·5	
Menopause	290	98·3	43	100·0	247	98·0	0·352
Supplements	258	87·5	37	86·1	221	87·7	0·762
Osteoporosis Medication	104	35·5	13	30·2	91	36·4	0·435
Bone density spine							0·281
Normal	45	15·9	10	23·8	35	14·5	
Osteopenia	99	35·0	12	28·6	87	36·1	
Osteoporosis	139	49·1	20	47·6	119	49·4	
Bone density femoral neck							0·273
Normal	41	14·4	9	21·4	32	13·2	
Osteopenia	147	51·6	22	52·4	125	51·4	
Osteoporosis	97	34	11	26·2	86	35·4	
Bone density total femur							0·480
Normal	80	28	15	35·7	65	26·6	
Osteopenia	161	56·3	21	50·0	140	57·4	
Osteoporosis	45	15·7	6	14·3	39	16	
Diagnosis							0·577
Normal	21	7·2	4	9·3	17	6·8	
Osteopenia	101	34·6	17	39·5	84	33·7	
Osteoporosis	170	58·2	22	51·2	148	59·5	

Data are presented as mean (sd) or median (IQR: Q1–Q3) or as number (percentage of total). The *P*-value indicates the statistical significance of the difference between nutritional background and no nutritional background groups. IQR, interquartile range.

### Item analysis

An English translated version of the administered question, the item difficulty and the discrimination values for the thirty items in the questionnaire are presented in Table 2. Item difficulty ranged from 0·13 to 0·96, with items 9, 23 and 29 having item difficulty > 0·90. No item had difficulty < 0·10. Item discrimination (R-value) showed medium correlations ranging from 0·211 to 0·455, and no item had an R-value < 0·200.

**Table 2.** Item analysis for the 30 items of the nutrition knowledge questionnaire

Item	Item difficulty (correct answers)	Item discrimination (R-value)
1 – How many servings (125 g) of milk and yogurt do experts recommend to consume each day?	0·33	0·251
2 – What is the average daily calcium requirement for adults?	0·17	0·297
3 – How many servings of cheese do experts recommend to consume weekly?	0·73	0·403
4 – How many grams is a recommended serving of fresh cheese?	0·42	0·341
5 – How many grams is a recommended serving of aged cheese?	0·72	0·350
6 – How does the nutritional requirement for calcium change in the older population compared to adults?	0·75	0·239
7 – What could be a good strategy to increase one’s calcium intake?	0·50	0·348
8 – Which of these components enhances calcium absorption?	0·84	0·230
9 – Which of these is a characteristic of the vitamin that enhances calcium absorption?	0·92	0·303
10 – Aside from calcium, which mineral is important for bone health?	0·36	0·282
11 – Which of these diets can worsen bone health?	0·44	0·359
12 – Why might a diet rich in fruits and vegetables be important for bone health?	0·83	0·276
13 – Which statement about mineral water is true?	0·66	0·365
14 – Is consuming two servings of 125 ml of milk per day sufficient to provide all the necessary calcium?	0·27	0·267
15 – Can a vegan diet never meet calcium requirements?	0·45	0·259
16 – Which of these foods contains the most calcium for the same weight?	0·19	0·211
17 – Which of these foods contains the most calcium for the same weight?	0·87	0·300
18 – How much calcium is in a serving of milk (125 ml)?	0·22	0·339
19 – How much calcium is in a serving of anchovies (150 g)?	0·13	0·322
20 – Compared to milk, soy beverages have:	0·45	0·280
21 – Which of these foods is a good source of vitamin D?	0·67	0·271
22 – In your opinion, having a diet with adequate consumption of milk and dairy products:	0·80	0·368
23 – In your opinion, being in menopause:	0·96	0·248
24 – In your opinion, smoking:	0·86	0·344
25 – In your opinion, being underweight:	0·58	0·455
26 – In your opinion, excessive alcohol consumption:	0·74	0·393
27 – At what age do you have the healthiest bones?	0·76	0·297
28 – Which of these is a risk factor for osteoporosis?	0·17	0·325
29 – Normally, who is at higher risk of osteoporosis?	0·92	0·338
30 – Which of these statements about the risk of osteoporosis is false?	0·66	0·434

### Validity and reliability of the questionnaire

The NK score showed a mean baseline score (t1) of 18·2 (sd 4·1), with nutritional/health background participants scoring significantly higher than no nutritional/health background participants (19·9 (sd 3·7) *v*. 17·9 (sd 4·1), *P* = 0·002), a difference that remained statistically significant after adjusting for educational level (*P* < 0·001). Follow-up scores (t2) averaged 18·6 (sd 4·1). Regarding test–retest reliability, Pearson’s correlation coefficient was R = 0·810 (95 % CI: 0·767, 0·850) and intraclass correlation coefficient was 0·806 (95 % CI: 0·760, 0·844), indicating strong temporal stability (Table 3).

**Table 3.** Psychometric evaluation of the nutrition knowledge score (*n* 295): construct validity, internal consistency and test–retest reliability

	Baseline Score (t1) *n* 295		Nutritional background (*n* 43)		No nutritional background (*n* 252)			Follow-up score (t2) *n* 295		Correlation		ICC		Cronbach’s *α*	
Score range	Mean	sd	Mean	sd	Mean	sd	*P*-value	Mean	sd	*r*	95 % CI	ICC	95 % CI	Standardized *α*	KR(20)
0–30	18·2	4·1	19·9	3·7	17·9	4·1	0·002	18·6	4·1	0·810	0·767, 0·850	0·806	0·760, 0·844	0·698	0·683

ICC, intraclass correlation coefficient (two-way mixed-effects model for multiple raters providing ratings for the same participants); Cronbach’s *α*, on initial (t1) responses; KR-20, Kuder-Richardson Formula 20 for binary items on initial (t1) scores. The *P*-value indicates the statistical significance of the difference between nutritional background and no nutritional background groups.

Internal consistency was good, with Cronbach’s alpha of 0·698 and Kuder–Richardson 20 of 0·683 at baseline (t1) (Table 3). Analysis showed that the overall reliability of the NK questionnaire remained stable with individual item removal, as indicated by standard Cronbach’s alpha and Kuder–Richardson 20 values close to the overall reliability metrics.

### Associations between nutrition knowledge score and other variables

Results of regression analyses indicate a significant negative association between NK score and age (F(1293) = 20·47, *β* = –0·146, se = 0·032, *P* < 0·001). Adjusting for age, the quadratic model showed a marginally better fit than the linear model, but this improvement did not reach conventional statistical significance (F(1292) = 2·96, *P* = 0·086; ΔRSS = 46·33). For BMI, NK score was negatively associated in a univariate model (F(1282) = 15·05, *β* = −0·193, se = 0·050, *P* < 0·001). When adjusting for age, both BMI (*β* = −0·164, se = 0·050, F(1281) = 15·39, *P* < 0·001) and age (*β* = −0·127, se = 0·033, *P* < 0·001) remained significant predictors. Adding a quadratic age term did not significantly improve model fit (ΔRSS = 35·08; F(1280) = 2·29, *P* = 0·132), indicating no additional explanatory power beyond linear age and BMI.

Regarding bone density T-scores, the spine T-score was marginally significant in predicting NK (F(1281) = 2·90, *β* = −0·332, se = 0·195, *P* = 0·090). In contrast, the femoral neck T-score showed a significant negative association when adjusted for age (*β* = −0·618, se = 0·246, F(1282) = 6·32, *P* = 0·0125). The relationship between the total femur T-score and NK score was best described by a quadratic function when controlled for age. The quadratic model provided a better fit than the linear model (Akaike Information Criterion for quadratic model: 1597·91 *v*. Akaike Information Criterion for linear model: 1614·98), and the quadratic term was statistically significant (F(1284) = 4·18, *P* = 0·042). In the quadratic regression, both the linear (*β*₁ = −1·163, se = 0·382, *P* = 0·003) and quadratic (*β*₂ = −0·298, se = 0·133, *P* = 0·025) components were significant (F(2283) = 4·699). The vertex of this quadratic curve, representing the highest point, was at the T-score for the total femur = −1·95 at NK score equal to 17·87.

## Discussion

A balanced diet contributes to the maintenance of a good health as well as for managing and preventing a wide range of medical conditions, including osteoporosis and related osteoporosis fractures. For this reason, understanding the level of NK and its association with dietary intake and bone health is essential^(27)^. This study led to the development of a NK questionnaire focused on the relationship between bone health and diet and to the assessment of its validity and reliability in a sample of 295 Italian women. Even though an initial cut-off of the difficulty index of 0·1−0·9 was established, based on a previous validation of another nutritional knowledge questionnaire^(13)^, three items with difficulty indices slightly above 0·90 were retained in the final questionnaire because they were considered as highly relevant by the expert panel for assessing fundamental knowledge of the topic. Thus, the final version of the questionnaire consists of thirty questions. Regarding the discrimination index of the items, all questions demonstrated medium-good correlation values (R > 0·02), showing that the questionnaire can correctly discriminate between people with higher and lower knowledge. Construct validity was assessed by comparing the total score of participants with nutritional/health backgrounds to those without such backgrounds. As anticipated, the group with a nutritional/health background was expected to have a NK score significantly higher than the group with no nutritional/health background. This difference remained statistically significant even after adjusting for educational level (*P* < 0·001), indicating that the questionnaire is capable of distinguishing between participants based on their disciplinary background independently of their overall educational attainment. Temporal stability was assessed using the test–rest method, wherein the questionnaire was administered at two separate times. The results demonstrated a high temporal stability (r = 0·810). The instrument also showed adequate overall internal consistency and reliability, with a Cronbach’s alpha of 0·698. While a cut-off of 0·7 typically indicates adequate internal consistency, some authors consider a Cronbach’s alpha value of exceeding 0·6 to be acceptable^(22,28,29)^. Notably, several publications have reported questionnaire validation with Cronbach’s alpha values ranging from 0·6 to 0·7^(13,30,31)^. The option to remove questions in order to increase internal consistency was not considered, as the overall reliability remained stable despite its removal; thus, all questions were retained to facilitate a more comprehensive investigation of the topics.

Due to the inability to perform external validation using other already validated tools, an association analysis was performed between NK and other known correlates. The results of the regression analysis indicated inverse associations between NK both with age and BMI. Previous research has reported that NK tends to decrease with increasing age and higher BMI, and this correlation is often attributed to several socioeconomic and psychological factors^(24,32,33)^. Older adults may have limited access to updated and truthful nutritional information, and they may rely on traditional dietary norms that are not aligned with current nutrition science, leading to misconceptions regarding healthy eating. Furthermore, higher BMI has been associated with lower NK, possibly because individuals with obesity may not have been exposed to effective nutrition education or may not prioritise healthy eating, regardless of age. Regarding bone health, a significant non-linear association with NK score and total femur T-score emerged, after adjustment for age. Although these findings are noteworthy, particularly in view of future research, they should be interpreted with caution and contextualised. Overall, these results emphasise the central role of age in shaping the associations between NK and bone health and indicate that, aside from the non-linear pattern observed at the total femur, the relationship between NK and bone health may vary across different levels of bone status. Comparison with the existing literature poses challenges due to the scarcity of studies addressing NK in relation to bone health among postmenopausal individuals. A recent study conducted on Iranian postmenopausal women, the major of whom had completed secondary school or high school, reported that women who received an educational intervention had higher BMD compared to those in the control group twelve months post-intervention^(9)^. This outcome may be explained by the fact that the nutrition education intervention, and the subsequent increase in NK, promotes preventive behaviours for osteoporosis among the participating women, such as adopting appropriate dietary practices and engaging in regular physical activity. Other studies also support the effect of educational interventions on lifestyle factors that improve BMD in this population group^(34,35)^, but none of them have specifically focused on improving nutritional knowledge. Dietary intake is one of the most important modifiable risk factors for osteoporosis and related osteoporotic fractures, and NK is a significant determinant of dietary intake, alongside factors such as taste, convenience, food cost or security, and cultural or religious beliefs^(27)^. Therefore, enhancing NK is crucial in prevention actions to change eating habits. Several studies have shown that increased NK leads to improved eating habits. A recent systematic review highlighted the positive association between BMD and adherence to the Mediterranean diet, reporting that an increase of only two points in the Mediterranean Diet adherence score (on a 9-point scale) was associated with a small increase in BMD at multiple sites, including the lumbar spine, femoral neck, hip, trochanter and whole body^(36)^. Moreover, several studies have shown that increased NK is associated with increased calcium intake^(10,37,38)^, which has proven to be essential for maintaining BMD and reducing the risk of osteoporosis, especially when combined with an appropriate vitamin D intake^(39,40)^. A recent study conducted in Italy observed an association between dietetic profile and NK, showing that individuals with a higher NK have nutritional habits more adherent to Italian food-based dietary guidelines^(41)^.

This study has limitations and strengths that should be highlighted. The validation of a NK questionnaire like NutriBone addresses several gaps in the literature. To the best of our knowledge, this is the first questionnaire on NK focused on the relationship between bone health and dietary behaviours that has been validated in Italy. While there are existing tools in the literature assessing knowledge of osteoporosis, such as OKAT^(42)^ and OKT^(16)^, they have not been validated at the Italian level. One of the limitations of the study is that the women aged 45–75 years were all recruited exclusively at the Osteoporosis Center of the Parma University Hospital among those women who were undergoing a DXA scan. This sample may have previously received information about preventive behaviours that positively influence bone health, either because of their age or because they have already been diagnosed with low bone mass and are therefore more interested in their bone health than the general population. Future studies should further investigate the link between NK and bone health, with particular attention to the key role of age, and including detailed assessments of dietary habits, nutritional status, physical activity and socioeconomic variables to provide a more comprehensive overview of this relationship.

The findings of the present study suggest that the NutriBone questionnaire is a reliable, valid and easy-to-use tool for assessing the NK related to bone health in Italian women undergoing DXA, regardless of their nutritional or health backgrounds. The NutriBone questionnaire could be used in future research to investigate the associations between nutritional knowledge, dietary habits and bone health and could be a starting point for the creation of similar tools for other population groups or countries.
